# Advances of Stem Cell Therapeutics in Cutaneous Wound Healing and Regeneration

**DOI:** 10.1155/2017/5217967

**Published:** 2017-10-29

**Authors:** Suman Kanji, Hiranmoy Das

**Affiliations:** ^1^Cardiovascular Stem Cell Research Laboratory, Wexner Medical Center, The Ohio State University, Columbus, OH, USA; ^2^Vascular Biology and Stem Cell Research Laboratory, Department of Biomedical Sciences, School of Pharmacy, Texas Tech University Health Sciences Center, Amarillo, TX 79106, USA

## Abstract

Cutaneous wound healing is a complex multiple phase process, which overlaps each other, where several growth factors, cytokines, chemokines, and various cells interact in a well-orchestrated manner. However, an imbalance in any of these phases and factors may lead to disruption in harmony of normal wound healing process, resulting in transformation towards chronic nonhealing wounds and abnormal scar formation. Although various therapeutic interventions are available to treat chronic wounds, current wound-care has met with limited success. Progenitor stem cells possess potential therapeutic ability to overcome limitations of the present treatments as it offers accelerated wound repair with tissue regeneration. A substantial number of stem cell therapies for cutaneous wounds are currently under development as a result of encouraging preliminary findings in both preclinical and clinical studies. However, the mechanisms by which these stem cells contribute to the healing process have yet to be elucidated. In this review, we emphasize on the major treatment modalities currently available for the treatment of the wound, role of various interstitial stem cells and exogenous adult stem cells in cutaneous wound healing, and possible mechanisms involved in the healing process.

## 1. Introduction

Skin, the largest organ of the body, has multiple important functions, such as acts as a barrier to foreign pathogens, regulates body temperature, supplies sensation, and prevents dehydration of the body [1]. An open wound could be defined as a type of injury in which the skin is torn, cut, or punctured resulting in disruption of normal anatomic structure and function [2]. Normal wound healing process is composed of a well-orchestrated process of cell migration, proliferation, and extracellular matrix deposition undergoing three overlapping but distinct phases of inflammation, proliferation, and maturation [3] and is a critical survival factor for an individual. Disruption of the cellular and molecular signals in conditions such as diabetes, infection, or radiation exposure may result in an inefficient healing. The skin wound might be of different nature and varies from surgical to accidental lacerations, burns, pressure ulcers, diabetic ulcers, and venous ulcers. In current medical practice, chronic cutaneous wound healing often demands a major, long-term medical attention and consumes a substantial amount of expenses [4]. The cost of treatment related to wounds and associated complications exceed $20 billion annually in the US [5]. For example, a diabetic foot ulcer typically costs around $50,000 to treat due to its refractory nature and continuous care [6]. Thus, enormous effort has been invested in developing innovative and efficient therapies to improve wound healing.

Current wound care has limited success and is very expensive. Thus, the approach of regenerative medicine has emerged as an alternative to improve the outcome of healing and has potential in reducing continuous economic burden. Regenerative therapy mainly focuses on stem cells that have the ability to self-renew and differentiate into multiple cell types and is crucial for physiologic tissue renewal and for regeneration after injury. As the understanding of stem cell biology grows through basic research, including preclinical models, stem cell-based therapies are increasingly evident in translational medicine. Current review emphasizes the understanding of the role of different endogenous and adult stem cells in cutaneous wound repair.

## 2. Events in Normal Wound Healing Process

The skin consists of three layers such as epidermis, dermis, and hypodermis. The epidermis, most outer layer, consists of multilayered epithelium extending from the basement membrane, which separates the dermis to the air. It is devoid of extracellular matrix (ECM) except the basement membrane. The basement membrane contains progenitor cells, which undergo continuous self-renewal and differentiate into keratinocytes. The keratinocytes migrate towards the surface of the skin where they eventually undergo terminal differentiation and maturation [3]. These keratinocytes form a keratinized layer of dead cells at the skin surface, which provides the main barrier [7]. The dermis is the thickest of the three layers of skin, which is present just below the epidermis. The dermis is a connective tissue comprised of fibroblasts, ECM, vascular endothelial cells, and skin appendages (hair follicles, sweat glands) [7]. Fibroblasts secrete molecules like collagen and elastin, which provide mechanical strength and elasticity to the skin. The hypodermis underneath the dermis is composed of adipose tissue, which provides insulation and cushioning between the skin and other skeletal structures, like bone and muscle [7]. Cutaneous wound healing process is imperative to restore a skin defect and to regain lost integrity, tensile strength, and barrier function of the skin [8]. Cutaneous wound repair is a multifaceted process involving inflammation, proliferation, and tissue remodeling [9].

### 2.1. Inflammation

**Figure 1:**
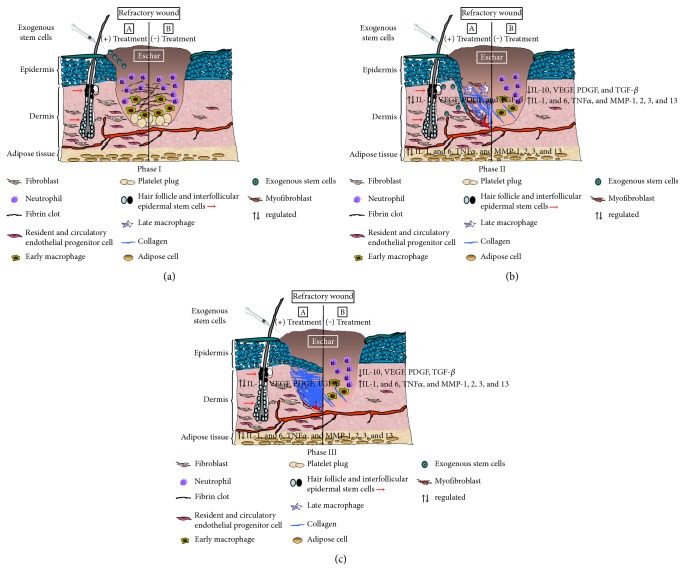
Graphical presentation of stem cell-mediated effect on refractory wound healing process. (a) Phase I: in inflammatory phase, the wound bed contains a large number of neutrophils, early phase macrophages, platelet plugs, and fibrin clots. Initiation of healing process occurs at this phase. (b) Phase II, A: systemic or local administration of stem cell homed to the wound bed. Exogenous stem cells mobilize host resident stem cells to take part in the healing process in GT formation by facilitating angiogenesis. Exogenous stem cells also directly take part in this healing process. The surrounding mobilized fibroblasts also differentiate into myofibroblasts and with collagen deposition facilitate reepithelialization process. B: in the absence of stem cell therapy, inflammatory cells such as neutrophils and macrophages still remain within the wound bed and impaired recruitment of endogenous stem cells occurs, which mediate an imbalance in the orchestrated harmony. GT formation is hindered due to the lack of angiogenesis, myofibroblast differentiation, collagen deposition, and reepithelialization. (c) Phase III: stem cell therapy generates scar tissue within the wound by replacing the provisional matrix. However, without stem cell therapy, refractory condition remains. The wound bed remains enriched with inflammatory cells and their proinflammatory secretory products. IL: interleukin; VEGF: vascular endothelial growth factor; PDGF: platelet-derived growth factor; TGF-*β*: transforming growth factor beta; MMP: matrix metalloproteinase.

The wound healing process starts with coagulation and fibrin clot formation called hemostasis. Platelets from damaged cutaneous blood vessels are exposed to ECM upon injury and damage. Fibrin binds to monocytes and neutrophils through integrin CD11b/CD18 receptor and participates in the inflammatory phase. Fibrin also binds to endothelial and fibroblast cells via *α*_v_*β*_3_ integrin [10] and stimulates angiogenesis. Platelets and mast cells release diffusible factors, such as tumor necrosis factor- (TNF-) *α* and platelet-derived growth factor (PDGF), and exert inflammatory response [11]. Local inflammatory agents, such as activated complement and histamine, cause redness and swelling. This matrix is rapidly invaded by neutrophils, followed by monocytes, and other immunocompetent cells to remove dead tissues and control infection. Polymorphonuclear cells (PMNs) are the first inflammatory cells to arrive at the site of a cutaneous wound in large numbers between 24–48 hours [12]. Several growth factors and cytokines, such as interleukin (IL)-8, PDGF, and growth-related oncogene (GRO)-*α*/CXCL1 chemokine (C-X-C motif) ligand, are involved in drawing PMNs to a wound bed [9]. These PMNs are the major source of proinflammatory cytokines, such as IL-1*α*, IL-1*β*, IL-6, and TNF-*α*, and exert cascades of inflammatory reactions and prevent infection (Figure 1(a)). PMNs are removed by macrophages through apoptosis, called PMN debridement via slough eschar [9, 12]. Monocytes come to the wound bed after PMN and transform into macrophages, which are abundant during day 2 and 3 but remain there for weeks. Different factors, such as macrophage chemoattractant protein- (MCP-) 1, macrophage inflammatory protein- (MIP-) 1*α*, vascular endothelial growth factor (VEGF), PDGF, and transforming growth factor- (TGF-) *β*, attract monocytes to the wound bed, and activated macrophages secrete IL-1*α*, IL-1*β*, IL-6, and TNF-*α* to perpetuate inflammatory reactions [9]. This inflammatory phase lasts for the first 4 days in normal wound healing process [13]. Besides eliminating microbes and debris, these inflammatory cells also initiate repair and mediate angiogenesis as the wound exits its inflammatory phase.

### 2.2. Tissue Remodeling

Inflammatory cells promote the recruitment and proliferation of fibroblasts, vascular endothelial cells, and keratinocytes during the proliferative phase [14]. Approximately 4 days after injury, the provisional ECM begins to be replaced by the granulation tissue (GT). GT is composed of fibroblasts, collagen, blood vessels, and macrophages. Fibroblasts are one of the most important cell types in the wound healing process. Several matrix metalloproteinases (MMPs), such as MMP-1, -2, and -3, play important roles in migration of fibroblasts into the provisional wound matrix. Fibroblasts secrete collagen, increased the amount of deposited collagens, especially collagen-I, and enhance cross-linking, which resulted in an increase in mechanical strength of the wound. Collagen production begins approximately 3 to 5 days after tissue injury and is stimulated by a number of growth factors, including PDGF, TGF-*β*, epidermal growth factor (EGF), insulin-like growth factor (IGF)-1, and fibroblast growth factor- (FGF-) 2 [9]. Fibroblasts differentiate into myofibroblasts, which promotes wound contraction and results in reduction of the wound area.

### 2.3. Proliferation

Neovascularization also occurs in concert with the help of invaded capillaries, recruited vascular endothelial cells, and endothelial progenitor cells to support the newly formed tissue and to transport circulatory cells to the wound [4]. Endothelial cell migration is initiated on day 2 of postwounding and stimulated by VEGF, FGF, angiopoietin, and TGF-*β*. Several MMPs including MMP-1, MMP-2, MMP-9, MMP-19, and membrane associated MT-MMPs play crucial a role in various aspects of angiogenesis. Deposition of GT mediates reepithelialization to the provisional wound bed. Keratinocytes migrate from the wound edges and proliferate on the surface of the GT [13]. For the progression of wound healing, bidirectional interactions between keratinocytes and fibroblasts are necessary by creating a paracrine loop [9, 15]. Occurrence of GT usually observed between 5 to 20 days of postwounding [7]. In the maturation phase, the wound becomes reepithelialized and the dermis regains most of its tensile strength. After complete wound closure, tissue remodeling takes place below the epidermis and may take up to a year or longer to complete [3]. In adults, a mature, nonerythematous flat linear scar formation is the hallmark of an ideal wound healing [16].

## 3. Acute and Chronic Wounds

Acute cutaneous wounds resulted from a trauma, which undergo a repair process and lead to a benign scar when the repair process is orderly and timely [2]. Failure of this process may lead to an undesirable scar or a nonhealing wound due to the extended wound area or the depth exceeds the patient's ability to heal (Figure 1). The ability to heal diminishes in different pathological conditions. Patients with chronic wounds (most notably diabetic foot ulcers) have underlying conditions, such as high blood sugar level and obesity, that impair wound healing. Pressure ulcers and venous ulcers are also some of the most common forms of chronic wounds. Chronic wounds are frequently linked to old age [17] and correlates with a poor reservoir of fully functional stem cells [18–20]. It is also linked with the age-related decreased strength and elasticity of skin and decreased blood flow to the extremities due to sedentary lifestyle and smoking [7]. Several studies suggest that psychological stress have a negative impact on wound healing [21, 22].

## 4. Current Treatments for Wound Healing

To achieve a complete healing of the wound, an appropriate wound care is critical, and standard treatment modalities are used to improve the wound bed. Therapy for chronic wounds mainly focuses on the identification and correction of the precipitating and perpetuating factors. This approach includes the use of antibiotics for accompanying cellulitis, revascularization of ischemic limbs, and compression devices for venous ulcers and rigorous off-loading for decubitus (pressure) ulcers [23, 24]. Despite the advancement in current wound care, chronic wounds do not heal or heal very slowly in the majority of the cases. Therefore, in recent years, efforts have been made to develop more and more advanced treatment strategies such as application of growth factors and cytokines [25], skin grafting [26, 27], and hyperbaric oxygen (HBO2) therapy [28].

### 4.1. Growth Factors and Cytokines

Therapeutic effects of various growth factors and cytokines were tested in the clinical management of nonhealing wounds. Among these growth factors, PDGF, VEGF, bFGF, and granulocyte-macrophage colony stimulating factor (GM-CSF) were tested extensively [29]. PDGF-BB was the most popular and approved by the Food and Drug Administration (FDA) for the treatment of diabetic neuropathic ulcers of the foot in the United States of America. However, later, the FDA announced the malignancy risk associated with this product [29, 30]. Hence, the journey of finding appropriate therapeutic growth factor for chronic wounds still continues.

### 4.2. Skin Graft

Efforts have also been devoted into tissue engineering in making appropriate skin grafts to heal refractory wounds successfully. The skin is the first tissue, which was successfully engineered in the laboratory for clinical application. There were two approaches to develop bioengineered skin, matrix-based product, where biodegradable matrix was used and the cell-based products, where cells were used for the application. There are bioengineered skin constructs, which are currently available and approved for clinical practice for the treatment of diabetic neuropathic ulcers. A bilayer living skin construct is also approved for venous and diabetic ulcers. Integra® is the first commercially available engineered skin substitute used for deep burn wound. Cross-linked collagen and chondroitin-6-sulfate copolymer are mixed together to form the dermal matrix. A silicon sheet is used which acts as a temporary epidermal layer [26]. Alloderm® is another skin substitute, specifically a dermal substitute, used for both wound repair and reconstructive surgery. This dermal substitute is made up of human cadaver dermis and used successfully for a full-thickness burn. Alloderm has reduced angiogenic components due to the risk of graft rejection [27]. Epicel™ is an example of a cultured autologous epidermis made up of human keratinocytes and used as an epidermal substitute for burned wounds, acute wounds, and chronic wounds [27]. Although Epicel has a little risk of rejection for large area wound coverage, this graft has limitation due to its short half-life and fragile nature. Instead of having novelty, artificially engineered skin is having certain disadvantages. In order to apply onto a patient, a skin biopsy not only takes several weeks to be expanded into sufficient cultured epidermis but also the product is very costly [26].

### 4.3. Hyperbaric Oxygen Therapy

Oxygen therapy under pressure also called as hyperbaric oxygen (HBO2) has been tried to improve wound healing for the last forty years with limited clinical benefits. HBO2 uses in wound healing on the basis of the fact that oxygen under certain pressure when applied to wounds can stimulate angiogenesis, promote fibroblast proliferation, and enhance immune function. There are very few evidences from clinical studies that demonstrate the efficacy of HBO2 therapy in any kind of foot ulcers or refractory wounds [28]. However, the application of HBO2 is currently not in clinical practice because this therapy could lead to significant side effects including myopia, oxygen toxicity in the brain leading to seizures, and pneumothorax [31].

Hence, approximately 50% of the patients with chronic ulcers do not heal when their ulcers were previously resistant to conventional therapy [32]. It is more and more evident from the wound healing experience of the last decade that more radical steps, such as stem cell therapy, need to be taken to propel the treatment of chronic wounds in a direction that will not only take care the external complexities of the wound but also will act on multiple modalities of wound healing systemically. For example, adult stem cells, which are multipotent and angiogenic, might be a suitable candidate for this purpose. Additionally, these stem cells can also be used as a vehicle for gene therapy, such as VEGF, and PDGF-BB [33, 34], which will add an extra dimension in treating chronic wounds such as diabetic ulcer. However, selection of a suitable cell type as a clinical candidate for wound healing therapy would be a great challenge.

## 5. Role of Stem Cells in Wound Repair (Endogenous and Exogenous)

The epithelium of the skin has a remarkable ability of self-renewal over the lifetime and also produces daughter cells that differentiate into one or multiple lineages. Cutaneous wound healing is the natural response but in case of severe conditions such as burn or diabetes, the repair process is insufficient to achieve an effective cure. In these chronic conditions, the result is neither aesthetically nor functionally perfect with the loss of epidermal appendages and the generation of connective tissue scar. Although epidermal stem cells in the basal layer, as an endogenous source of stem cells, can regenerate skin, but these cells are not sufficient to provide perfect repair after deep and extensive skin damage. Thus, exogenous supply of stem cells in traumatic conditions may be one of the novel therapeutic strategies to achieve perfect skin repair.

## 6. Endogenous Stem Cells

### 6.1. Hair Follicle and Interfollicular Epidermal Stem Cells

Three major compartments of the epidermis, such as interfollicular epidermis, sebaceous gland, and hair follicle, are capable of self-renewal (Figure 1). Among these compartments, interfollicular epidermis and sebaceous glands undergo constant self-renewal, whereas hair follicles undergo cycles of phases such as resting, growth, and involution [35]. In physiological condition, these compartments of the epidermis are rejuvenated by the differentiation of their own stem cells. However, during injury, these epidermal compartments are capable of repopulating one another [36, 37]. In case of full-thickness wounds, where the hair follicle is obliterated, wound healing occurs slowly from the wound edge; whereas, in case of partial thickness, wound healing is accelerated and relies on reepithelialization with the migration of cells from the hair follicle and sebaceous gland [38, 39]. The hair follicle bulge to epidermal stem cells in partial thickness wound regeneration, which is transient and bulge-derived cells are replaced eventually by interfollicular epidermal stem cell progeny as the injury is recovered or stress is relieved [37, 40]. Although bulge epidermal stem cells are not essential for wound closure [39], these cells significantly expedite closure in the early stages of wound healing [41]. Thus, hair follicle and its connective tissue sheath are attractive targets for the development of regenerative therapies due to its accessibility and richness of stem cells.

### 6.2. Endothelial Progenitor Cells

Endothelial progenitor cells play an important role in wound healing process via angiogenesis and facilitate wound closure. These progenitor cells might be tissue resident or originate from the bone marrow. Bone marrow-derived endothelial progenitor cells home to the site of cutaneous injury in response to hypoxia-inducible factor (HIF)-1-induced stromal cell-derived factor (SDF)-1 in hypoxic milieu [42, 43]. However, these phenomena are impaired in pathophysiological conditions such as diabetes and with the age [44]. Thus, diabetic wound fails to heal, especially with the increasing age. Hence, appropriate exogenous stem cell transplantation might be an alternative strategy to cure chronic wounds. It is also believed that resident endothelial progenitor cells in the skin also contribute to wound neovascularization through angiogenesis [45]. Isolated tissue resident endothelial progenitor cells contribute to angiogenesis by differentiating into blood vessels upon transplantation [46].

## 7. Cell-Based Therapy for Wounds

Human stem cells may offer considerable opportunities providing both undifferentiated and differentiated cells for gene therapy, drug discovery, and regenerative medicine [47]. In addition, stem cells could be transduced ex vivo and manipulated cells reintroduced into the host. Manipulated stem cells could also offer new therapeutic approaches for specific diseases conditions. Wound repair is a complex process and is influenced by numerous secreted factors, including cytokines, chemokines, and growth factors. In theory, application of stem cells to wounds is advantageous over administration of a single agent because stem cells have a unique feature of interacting with wound environment and modulate their activity to release multiple factors, which may facilitate wound healing process (Figure 1). Stem cells can also potentially serve as a source of cells for providing skin substitutes in applications for tissue engineering. Thus, the selection of a suitable stem cell is a challenge in order to achieve a desirable efficacy in wound healing. Embryonic stem cells could be the most favorable over adult stem cells for the repair and regeneration of skin tissues due to their capacity of self-renewal and unlimited supply of differentiated keratinocytes or keratinocyte progenitors for treating cutaneous injuries. However, embryonic stem cell-related research has raised difficult ethical issues and has evoked a great public interest and controversy.

Moreover, embryonic stem cells have a potential to generate tumors. The development of therapies using stem cells in the context of injury and wound healing has primarily relied on adult stem cells. Adult stem cells derived from the bone marrow, peripheral blood, umbilical cord blood, or adipose tissue with their limited capacity of self-renewal and proliferation would be more acceptable for therapeutic application in human skin tissues. Thus, an immense amount of research is going on to prove the efficacy and mechanisms of action of these stem cells for skin regeneration. There are already some encouraging results from human studies using multipotent adult stem cells as therapeutic agents for tissue repair [44, 48–50]. Endogenous stem cell populations are thought to play an important role in different aspects of skin wound healing including inflammation, reepithelialization, neovascularization, and tissue remodeling [51]. However, in pathological conditions, it has been observed that administration of exogenous adult stem cell accelerated wound healing through various mechanisms such as acceleration of reepithelialization, stimulation of neovascularization in a paracrine manner, or directly differentiating into various cell types such as keratinocyte, fibrocytes, endothelial cells, and pericytes (Table 1).

### 7.1. Embryonic Stem Cells

Embryonic stem cells (ESCs) are pluripotent in nature which reside within the blastocyst. These cells have a potential to differentiate into any of the three primary germ layers namely endoderm, mesoderm, or ectoderm [52]. Embryonic stem cells can be differentiated into keratinocytes in presence of selected medium containing specific growth factors. These keratinocytes are capable of forming multilayered epidermis in culture, making them a key cell type for bioengineered skin [53]. However, the use of embryonic stem cells remains controversial, as ethical concerns exist regarding the harvest of cells from live embryos. Moreover, the potential for immune rejection and teratoma formation remains as other concerns. Hence, focus has been redirected towards adult stem cells as an alternative source with potential to apply in various disease conditions.

### 7.2. Induced Pluripotent Stem Cells

Induced pluripotent stem cells (iPSCs) are the multipotent cells with self-renewal properties, which are engineered from differentiated adult somatic cells, such as fibroblasts and keratinocytes, using transcription factors (e.g., Oct-3/4, Sox2, c-Myc, and KLF4) [54–56]. Unlike ESCs, iPSCs not only eliminate ethical issues but also reduce the chances of immune rejection while using it therapeutically [57]. A negligible immune response was also observed in iPSCs derived from human skin fibroblasts [58]. The unique reprogramming of iPSC technology made it possible to generate genetically diverse patient-specific cell lines from genetic skin disorders or chronic wounds which have tremendous potential for disease modeling and drug screening [59, 60]. During the last decade, significant progress has been made in the differentiation of the mouse, human iPSCs in to dermal stem cells and hair follicle lineages [58, 61], mesenchymal cells with the potential of forming dermal papilla [62], fibroblasts [63], melanocytes [64], keratinocytes [65, 66], among others. The multipotent capacity with limited immunoreactivity of iPSCs makes them a prospective agent for treating chronic skin disorders and unresolved wounds [67]. iPSCs generated from patients also could be modified and have the potential for cell therapy that have been shown in several studies as a proof of concept [66, 68]. However, application of iPSCs in human patients need further extensive analyses for safety and reliability of the reprogramming technology due to the risk of teratogenicity, mutagenesis, among others [69]. iPSCs can also provide a foundation for modeling a complex human organ like skin tissue due to their ability to be differentiated into multiple cell types in the body, and their unlimited growth potential was also demonstrated in various *in vivo* models [70, 71]. iPSCs therefore hold a great promise in the field of wound repair and regenerative medicine.

### 7.3. Mesenchymal Stem Cells

Mesenchymal stromal cells, also known as mesenchymal stem cells (MSCs), are adult stem cells capable of self-renewal and multipotential differentiation [72, 73]. MSCs can be obtained from the bone marrow and other tissues such as adipose tissue, nerve tissue, umbilical cord blood, and dermis with phenotypic heterogeneity [74–79]. In regenerative medicine, unlike embryonic stem cells, the use of mesenchymal stem cells could avoid ethical issues. Also, allogeneic MSC transplantation may induce little immunoreactivity to the host [80, 81]. Thus, MSCs have received considerable attention for modulating wound repair [82]. MSCs have been tested for skin repair and regeneration in various acute and chronic skin injuries like acute incisional and excisional wounds, diabetic skin ulcers, radiation, and thermal burns [76, 83, 84]. Inflammation and oxidative stress generated during wound healing not only attract bone marrow-derived mesenchymal stem cells at the wound area and conducive to self-renewal and proliferation [85] but also support wound healing through differentiation and the promotion of blood vessel formation. MSC therapy has shown enhanced wound healing through increased angiogenesis, reepithelialization, and tissue granulation. In clinical settings, MSC also showed a great promise in treating refractory wounds. In clinical studies, after MSC treatment, patients showed improvement of their wounds within days following administration, characterized by a decrease in wound size, an increase in the vascularity of the dermis, and increased dermal thickness of the wound bed [48, 86]. Additionally, coadministration of MSC at the wound site along with an autologous graft composed of autologous skin fibroblasts on biodegradable collagen membranes also decreased wound size and increased vascularity and dermal thickness in chronic diabetic foot ulcers [84]. All these findings from preclinical and clinical studies demonstrated that MSCs can contribute to wound repair and may be a resource for regenerative therapy.

### 7.4. Adipose-Derived Stem Cells

Adipose-derived stem cells (ASCs) are the precursor cells that are present within the stromal-vascular fraction of an enzymatically digested fat tissue. Minimal invasive nature of tissue harvest has made these stem cells more attractive for regenerative medicine. ASCs are multipotent in nature and can be differentiated into different lineages such as bone, fat, cartilage, and muscle [75, 87]. ASCs can be characterized while in culture dish as CD73^+^/CD90^+^/CD105^+^/CD44^+^/CD45^−^/CD31^−^ cells, which can be distinguished from the bone marrow-derived MSCs by their expressions of CD36 and negative for CD106 molecules on their cell surface [88]. Although both of these cell types share surface markers, biologically they are different in terms of proliferation rate and differentiation, cytokine secretion, and chemokine expressions [89–91]. Thus, ASCs and MSCs may contribute to the wound healing differently. The capability of ASCs to secrete growth factors, to differentiate into multiple cell types, and to promote angiogenesis renders them a viable skin substitute [92, 93]. The ability of ASCs for soft tissue reconstruction makes them attractive for wound healing [94].

### 7.5. Hematopoietic Stem Cells

The possible role of hematopoietic stem cells (HSC) in skin regeneration is evident in many occasions. HSC can be isolated from the bone marrow (BM), umbilical cord blood, and peripheral blood by using its surface markers. In several occasions, skin “chimerism” (identification of epithelial cells of donor genotype) has been observed after clinical HSC transplantations such as BM or peripheral blood mononuclear cells (PBMC) [95–97]. The findings of donor-derived contribution of HSC to epithelial lineages in the host offer the broad-spectrum plasticity of HSC and indicate the possibility of skin regeneration by transplantation of HSC in chronic wound disorders. In a murine excisional wound model, a significant number of differentiated green fluorescent protein (GFP) positive cells were found in the hair follicles, sebaceous glands, and epidermis in host skin 21 days after transplantation of syngeneic GFP + bone marrow cells [48]. Additionally, a study has also shown that the differentiation potential of human umbilical cord blood stem cells into keratinocytes *in vitro* [98]. Apart from plasticity, the role of HSC in angiogenesis is also evident in myocardial infarction model, which is important and may be ascribable for the perfect and functional repair of skin tissue [99]. An emerging concept, epithelial and mesenchymal cell interaction is supposed to be a vital phenomenon in keratinocyte proliferation and differentiation, might play a crucial role in cutaneous wound healing and reepithelialization [4, 92]. The expression of CD34 and CD133 cells in dermal fibroblast and follicular matrix during embryogenesis provides an indication for the role of HSC in the molecular control of epithelial-mesenchymal cell interactions [100].

Peripheral blood, fetal aorta, and umbilical cord blood are also enriched with stem and progenitor cells, which express CD34 and CD133 markers. These cells are also multipotent and have shown a neovascularization potential in preclinical ischemic models [33, 34]. In preclinical wound healing models, we and others reported that CD34+ or CD133+ cells accelerate wound closure. We have demonstrated the wound healing ability of nanofiber-expanded cord blood-derived CD34+ cells in a mouse excisional wound model and an *in vitro* cellular model. We have shown that after systemic administration, these stem cells reached to the wound bed and facilitated wound healing. Our study revealed that nanofiber-expanded cord blood-derived CD34+ cell therapy accelerates wound healing by inhibiting several matrix metalloproteinases at the wound bed which prevents collagen degradation and increased the abundance of collagen components, procollagen1A1 at the wound bed [101]. Unlike previous experiments, we demonstrated for the first time that nanofiber-expanded cord blood-derived CD34+ stem cells accelerated wound closure by secreting collagen and thereby positively contributed to extracellular matrix [101], indicating that CD34+ stem cell treatment is having a potential to treat the refractory wounds resulting from diabetes or traumatic skin injuries. We further extended this work to explore the regulation of inflammatory response by CD34+ cell therapy using the same mouse wound model. Overall, our study demonstrated that CD34+ cell therapy mediated suppression of prolonged inflammation, positively contributed to increased angiogenesis, and accelerated wound closure compared to nontreated wounds [102]. These data provided a valuable information regarding the benefits of CD34+ stem cell-mediated wound healing and cell therapetic mechanism behind accelerated wound closure. In another study, treatment with human CD34+ peripheral blood mononuclear cells also accelerates healing of full-thickness skin wounds in diabetic mice by accelerated revascularization and epidermal healing [103]. A similar observation was also found in a report where cord blood-derived CD34+ cell treatment accelerated diabetic wound closure by stimulating keratinocytes, fibroblast proliferation, and neovascularization in a paracrine manner [104]. In another study, human fetal aorta-derived CD133+ progenitor cells and their conditioned medium treatment accelerated healing in ischemic diabetic ulcer by stimulating angiogenesis with activation of the Wnt signaling pathway in the host [105]. These findings indicate that blood-derived progenitors may have a therapeutic potential in the treatment of skin lesions in complex pathological conditions such as diabetes.

## 8. Mechanisms of Stem Cell-Mediated Wound Healing

### 8.1. Immunomodulation, Resolution of Inflammation, and Fibrosis

An imbalance in regulation of inflammation at the wound bed leads to defective healing. Sustained unresolved inflammation leads to chronic wound. Prolonged inflammation even leads to fibrotic scar formation. In chronic wounds, unresolved inflammation leads to increased protease activity and deregulated fibroblast activity, which resulted in decreased collagen deposition and ECM formation. Hence, resolution of inflammation is a big challenge in diabetic wound healing. In recent years, several studies have demonstrated the immunomodulatory function of cultured adult stem cells in laboratory conditions obtained from various sources like the umbilical cord blood, amniotic fluid, and bone marrow. Thus, allogeneic stem cell therapy induces immunomodulation in the wound bed and facilitates wound healing by resolving inflammation, as well as helping in reducing scar formation [106].

A substantial number of studies have demonstrated that treatment of MSCs has significant immunomodulatory effects during wound healing and in other inflammatory conditions [107]. This immunomodulatory effect on the host not only makes them a suitable candidate for allogeneic transplantation [108] but also makes them an attractive cell therapeutic agent to treat chronic wounds [106]. Studies have demonstrated that MSCs obtained from various sources such as the umbilical cord and bone marrow showed an anti-inflammatory effect in rat cutaneous wound and *in vitro* fibroblast model. MSC treatment demonstrated a significantly lower number of inflammatory cells and proinflammatory cytokines such as IL-1 and TNF-*α* with an increased level of IL-10 at the cutaneous wound bed in a rat model [109]. In addition, when murine BM-MSC were cocultured with human fibroblasts, the mRNA levels of intercellular adhesion molecule 1 (ICAM1) has decreased [110]. These studies suggest the potential of MSC in attenuating wound inflammation and inducing healing in chronic inflammatory stage.

Allogeneic transplantation of cord blood and cord blood-derived stem cells is also regarded as less immunogenic [111]. Our results demonstrated that nanofiber-expanded cord blood-derived CD34+ cells might have an immunomodulatory effect *in vitro* and *in vivo* wound healing models [102]. Systemically transplanted CD34+ cells accelerated wound closure, which was correlated with decreased inflammatory activity at the wound bed characterized by reduced inflammatory gene expression such as, IL-1*β*, TNF-*α*, IL-6, and NOS2A. At the same time, expression of anti-inflammatory molecule IL-10 was significantly increased indicating that CD34+ cell therapy has the potential to control the inflammation during wound healing process. To further elucidate the mechanism, we showed that CD34+ cells secrete IL-10, an anti-inflammatory molecule, and suppress NF-*κ*B activation in a human primary fibroblast cell model. In the similar *in vitro* model, when human primary fibroblasts were cocultured with CD34+ cells in presence of inflammatory stimulus with TNF-*α*, NF-*κ*B activation was significantly decreased by upregulation of IL-10 [102]. Sustained or unresolved inflammation prevents wound healing by inhibiting angiogenesis and catabolizes extracellular matrix in the wound bed. Our results suggest that nanofiber-expanded cord blood-derived CD34+ cell therapy might be a potential candidate to treat chronic wounds to resolve inflammation in a timely manner that will facilitate further angiogenesis and ECM formation for accelerated healing. Other studies have also suggested that ASCs also modulate the immune system and downregulate the inflammation by releasing growth factors critical for healing which are described in the reviews [112].

Antimicrobial activity is critical for wound clearance from infection. Studies have shown that MSCs have antimicrobial activities, which may also be helpful for chronic wound resolution. Antimicrobial activities of MSCs were shown directly by the secretion of antimicrobial factors such as LL-37 [113]. In another study, it was shown that MSCs could secrete immune-modulating factors, which will upregulate bacterial killing and phagocytosis by immune cells [114].

Tissue resident and peripheral macrophages play a significant role in initiation of inflammation after injury and resolving inflammation in a timely manner during the healing process. Macrophages shift gears between proinflammatory M1 and anti-inflammatory alternatively activated M2 states. Studies have shown in various models that MSCs may influence macrophage M1/M2 polarization after contact with macrophage [115–117]. M2 polarized macrophages play an important role in the resolution of inflammation and clearance of dead cells from the wound environment for accelerated healing. In a mouse wound healing model, it was shown that the human gingiva-derived MSC treatment *in vivo* promoted an M2 macrophage polarization, which was correlated well with anti-inflammatory wound environment and accelerated cutaneous wound healing [118]. The ability of MSCs to resolve inflammation might be useful in treating chronic unresolved wounds.

Moreover, dysregulated fibrosis or scarring is caused by excessive deposition of ECM. Inflammation largely regulates fibrosis process. The immunomodulatory activity of MSCs might regulate fibrosis and therefore anti-inflammatory activity of MSCs reduces the scar formation. In an *in vivo* murine wounding model, it showed that BM-MSCs attenuated development in skin fibrosis [119].

### 8.2. Differentiation

In several studies, it has shown that adult stem cells are multipotential and able to contribute to wound healing by differentiating into several tissue lineages starting from the inflammatory cells to myofibroblasts. During the inflammatory phase, HSCs from the bone marrow undergoes myelopoiesis with the help of MSC and supply the leukocytes to the wound region [92]. Also, tissue resident stem cells undergo differentiation in response to various stimuli during the wound healing process [120, 121]. From the regenerative perspective, differentiation is one of the key phenomenon by which exogenously applied adult stem cells exert therapeutic efficacy in cutaneous wound models. MSC is one of the most widely studied in this regard. Several studies have demonstrated that transplanted MSCs can differentiate into epidermal keratinocytes, endothelial cells, and pericytes directly participating in the structural repair of a wound. MSC transplantation led to accelerated cutaneous wound closure in both normal and diabetic mice, where MSCs express keratinocyte-specific markers suggesting their role to promote wound healing by differentiation [76]. Similarly, another study demonstrated that MSCs also transdifferentiate into keratinocytes, endothelial cells, and pericytes in cutaneous wounds after intravenous injection in mice [122]. Adipose-derived stromal cells are also capable of differentiating into epithelial, endothelial, and fibroblast lineages *in vivo* when applied to wounds by means of a seeded scaffold [92]. A similar kind of low-level transdifferentiation phenomenon was also observed in human skin after HSC transplantation where the mesodermal origin of HSC contributes to epithelial lineage in the host [95, 96].

### 8.3. Angiogenesis

Optimal repair and restoration of functional vasculature is crucial to achieve ideal healing of wounds. The process of neovascularization primarily accomplishes revascularization of the wound bed. Neovascularization is achieved through two independent processes called angiogenesis and vasculogenesis, which lead to the development of functional microvascular networks. Traditionally, vasculogenesis is the de novo synthesis of new vessels by endothelial progenitor cells whereas angiogenesis is the development of new vessels from existing capillaries [123]. In a refractory wound-like diabetic wound, wound revascularization is affected due to an imbalance in the release of soluble mediators and improper function of endogenous progenitor and stem cells which is essential for ideal wound healing, leading to a hindered and orchestrated healing process. Several other and our own published studies [101, 102] have demonstrated that transplantation of adult stem or progenitor cells contribute to angiogenesis or vasculogenesis through directly differentiating into cell types essential for blood vessel formation or by stimulating endogenous mediators or cells in a paracrine manner. Studies have found that MSC expresses high levels of vascular endothelial growth factor (VEGF) and angiopoeitin-1, which indicate that the MSC-mediated accelerated wound healing is due to the release of proangiogenic factors and induction of angiogenesis [76]. Additionally, paracrine signaling and the release of soluble factors (e.g., VEGF) by MSC are found to promote angiogenesis at the wound bed after cutaneous injury in normal and diabetic mice [124, 125]. Another interesting hypothesis about MSC is that these cells may also act as pericytes, which stabilizes the blood vessel formation [126]. Future studies will further bolster this claim. However, the role of MSC in promoting angiogenesis is firmly evident by several instances in normal and refractory wound healing models [127, 128].

In other stem cells like ASC, it was noticed that ASC treatment also promotes angiogenesis and accelerates wound healing by producing VEGF [93]. Thus, induction of angiogenesis to accelerate wound healing by stem cell therapy is not only evident in MSC and ASC but is also found in several other types of stem and progenitor cells [103, 105]. Thus, increased angiogenesis by stem cell therapy not only supports GT formation but also supplies nutrients and clear the apoptotic cells from the wound bed and helps in wound resolution.

## 9. Conclusions and Future Directions

It is evident that stem cells have a tremendous potential for cutaneous tissue regeneration, as these cells not only can regenerate lost tissue but also promote wound repair through paracrine manner. Several cell types, such as embryonic stem cells, iPSCs, mesenchymal stem cells, resident tissue stem cells, epithelial stem cells, adipose-derived stem cells, and hematopoietic stem cells, are currently under intense investigation. Recent data on autologous MSC therapy in cutaneous repair showed a great promise as a therapeutic agent in clinical practice. Despite rapid progress in evaluating the efficacy of MSC transplantation for wound healing, several questions still need to be addressed. Using specific markers to characterize and isolate a distinct pool of MSC, which is homogeneous and functional, is one of the key points to be addressed. Further studies are necessary to characterize the niche of MSC, which helps MSCs to be effective in the wound healing process. Further investigation on experimental and clinical application of stem cells in wound healing is necessary to identify the ideal source of stem cells and the most efficacious mode of cell delivery.

Human umbilical cord blood is rich in stem and progenitor cells and is easily accessible for blood collection. The regenerative potential of CD34+ and CD133+ cell therapy in cutaneous tissue repair obtained from peripheral or umbilical cord blood opens up a possibility of providing cheap and affordable care for refractory wounds. Thus, a suitable technology like ex vivo expansion technique would be useful to get a large number of stem cells for the clinical application. Our group and others have shown promising results in expansion of umbilical cord blood-derived stem cells. The expanded stem cells were characterized (CD34+) and have shown their multipotential and angiogenic capabilities in preclinical ischemic models as well as in murine cutaneous wound models [33, 34, 101, 102] where CD34+ stem cell therapy accelerated wound closure by resolving inflammation with concurrent inhibition of MMP expressions. Additionally, these cells also can be manipulated *in vitro* with proangiogenic factors like VEGF and PDGF, which are efficacious in improving ischemia-related complications in preclinical peripheral and cardiac ischemic models. Thus, looking at their angiogenic and anti-inflammatory potential, this pool of stem cells may have a very promising future in treating refractory wounds. Moreover, nanofiber-expanded stem cells coupled with the innovative biotechnologies may open a new direction for plastic and reconstructive surgeons. Finally, the use of stem cells to induce cutaneous tissue regeneration holds a great promise for modern regenerative medicine.

## Figures and Tables

**Table 1:** Overview of stem cell-based therapies for cutaneous wound management *in vivo* and their mechanism of action.

Wound model	Type of cell therapy	Regenerative mechanisms	Reference
Excisional wound splinting model/Balb/C mice	BM-MSC	Recruitment of macrophages and endothelial lineages by paracrine factors	[124]
Excisional wound splinting model/diabetic mice	BM-MSC	Differentiation and angiogenesis	[129]
Excisional wound splinting model/C57BL/6 J mice	GMSC	Immunomodulation, M2 macrophage polarization	[118]
Full-thickness excisional wound/STZ-induced diabetic rat	ASC	Differentiation and vasculogenesis	[130]
Full-thickness excisional wound/STZ-induced diabetic mice	Human blood-derived CD34+ cells	Vasculogenesis	[103]
Limb ischemia and wounding/STZ-induced diabetic mice	Human fetal aorta-derived CD133+ cells	Paracrine stimulation of angiogenesis and activation of Wnt signaling	[105]
Full-thickness excisional wound in NOD/SCID mice	Nanofiber-expanded cord blood-derived CD34+ cells	Fibroblast proliferation and enhancement of collagen deposition and decreased MMP expression	[101]
Full-thickness excisional wound in NOD/SCID mice	Nanofiber-expanded cord blood-derived CD34+ cells	Immunomodulation, angiogenesis	[102]

BM-MSC: bone marrow-derived mesenchymal stem cells; GMSC: human gingiva-derived MSC; STZ: streptozotocin; ASC: adipose-derived stem cells.
